# Insight Into Mouse Models of Hyperthyroidism

**DOI:** 10.3389/fendo.2022.929750

**Published:** 2022-06-22

**Authors:** Mengyu Zhang, Wen Jiang, Ganghua Lu, Ru Wang, Zhongwei Lv, Dan Li

**Affiliations:** ^1^Department of Nuclear Medicine, Shanghai Tenth People’s Hospital, Tongji University School of Medicine, Shanghai, China; ^2^Imaging Clinical Medical Center, Tongji University School of Medicine, Shanghai, China; ^3^Clinical Nuclear Medicine Center, Tongji University School of Medicine, Shanghai, China

**Keywords:** hyperthyroidism, graves’ disease, autoimmunity, levothyroxine, treatment

## Abstract

Hyperthyroidism is characterized by an increase in the synthesis and secretion of thyroid hormones in the thyroid gland, and the most common cause of overproduction of thyroid hormones is Graves’ disease (GD). Long-term disease models of hyperthyroidism have been established. In general, methods to induce GD include transfection of fibroblasts, injecting plasmids or adenovirus containing thyroid stimulating hormone receptor (TSHR) or TSHR subunit, and exogenous artificial thyroid hormone supplementation. Fortunately, in mouse studies, novel treatments for GD and Graves’ orbitopathy (GO) were discovered. It has been reported that prophylactic administration of TSHR A subunit protein in genetically susceptible individuals could induce immune tolerance and provide protection for the future development of GD. Biologically active monoclonal antibody against intracellular adhesion molecule-1 (ICAM-1 mAb) and siRNA targeting TSHR can also be used to treat GD. Moreover, new potential therapeutic targets have been identified in GO mouse models, and these targets could present novel therapeutic approaches. Besides, human placental mesenchymal stem cells (hPMSCs) into the orbit, fucoxanthin and icariin may be new alternative therapies that could be used in addition to the existing drugs, although further research is needed.

## Introduction

Hyperthyroidism is a characteristic clinical condition caused by excessive thyroid hormone concentrations in tissues (1). The common causes of hyperthyroidism includes Graves’ disease (GD), toxic nodular goiter, painless thyroiditis or thyroid dysfunction induced by drugs (2). The common clinical manifestations of hyperthyroidism are palpitations, tremor, heat intolerance, sweating, polydipsia and weight loss caused by hypermetabolism (1). Patients with long-term untreated hyperthyroidism may even develop atrial fibrillation or heart failure (3). Thus, exploring the pathogenesis of hyperthyroidism and seeking effective treatment are of great importance.

Various mouse models have been used to investigate hyperthyroidism (4). Researchers have carried out immunization approaches, such as injecting the adenovirus or plasmid accompanied by thyroid stimulating hormone receptor (TSHR), or chemical methods, such as levothyroxine supplementation (5–7). Moreover, from studies using the above modelling methods, we found that the establishment of immune tolerance can provide protection against the further development of GD (8, 9). Additionally, targeted therapy is also a promising new treatment for GD (10).

In this review, we summarize the research progress of mouse models of hyperthyroidism and the potential novel treatments for GD and GO that have been identified using these models. This summary helps to better understand the pathogenesis of hyperthyroidism, reduce the incidence of hyperthyroidism and provide a potential therapeutic strategy for hyperthyroidism.

## Murine Model of Hyperthyroidism Induced by TSHR

GD is the most common type of hyperthyroidism. The mouse models of GD are mainly achieved by inducing the expression of TSHR *in vivo* (**Table 1**).

**Table 1:** Summary of mouse models of hyperthyroidism.

Protocol	Species	Hyperthyroidism	Reference
**Injection of cells transfected with TSHR**			
Fibroblasts transfected with TSHR, 6 times	Female AKR/N	Low incidence	(11)
Fibroblasts transfected with TSHR, 8 times	Female AKR/N	35%	(12)
**Plasmid encoding TSHR**			
Intramuscular injections, 3 times	Female NMRI	20%	(13)
Intramuscular injections, 3 times	Female BALB/c	None	(14)
**Adenovirus containing TSHR or TSHR-289**			
Intramuscular injection of TSHR	Female BALB/c	More than 50%	(15)
Intramuscular injection of TSHR	DBA/2J	5%	(15)
Intramuscular injection of TSHR-289, 3 times	Female BALB/c	High incidence	(16)
Intramuscular injection of TSHR-289, once	BALB/c	High incidence	(17)
**Exogenous levothyroxine supplementation**			
Intraperitoneal injection of levothyroxine, 28 days	C56BL/6	High incidence	(18)
Oral levothyroxine, 6/7 weeks	C57BL/6NTac	High incidence	(19)

GD models can be induced by injecting cells that stably express TSHR into mice. Shimojo et al. injected female AKR/N mice with fibroblasts transfected with cDNAs for human TSHR and MHC class II molecules. After six injections, most mice developed TSHR antibodies characterized as GD (11), and this was replicated in other studies (12, 20).

After many attempts, other methods of inducing GD were also discovered. Many studies have induced GD by immunization of plasmids or recombinant adenovirus vectors that express TSHR instantaneously *in vivo*. Costagliola et al. used a plasmid that encodes TSHR for genetic immunization, which induced Graves’-like hyperthyroidism (13). However, only 20% of the female NMRI mice immunized with TSHR cDNA developed hyperthyroidism and thyroid stimulating hormone receptor-stimulating antibody (TSAb), and none of the male NMRI mice developed hyperthyroidism in their experiment (13). There were even studies showing that hyperthyroidism was not even observed in BALB/c mice immunized by intramuscular injection of an expression vector constructed from human TSHR cDNA (14).

Moreover, Nagayama et al. established a new mouse model of GD by injecting recombinant adenovirus that expresses thyrotropin receptor (15). Over 50% of the female BALB/c mice developed hyperthyroidism, but only 5% of the DBA/2J mice developed hyperthyroidism (15). This suggests the role of genetic factors in the susceptibility to Graves’ hyperthyroidism in mice. A growing number of experiments have confirmed this hypothesis, with many studies demonstrating that BALB/c mice are susceptible to GD in the TSHR-adenovirus model (21). In order to further improve the success rate of inducing hyperthyroidism, many researchers have used an adenovirus containing TSHR-289, a subunit encoded human TSHR, to induce GD (16, 22). Studies have proven that the proportion of hyperthyroidism induced by adenovirus carrying TSHR-289 was significantly higher than that of full-length wild-type adenovirus containing TSHR (23). Besides, Tang et al. immunized BALB/c female mice with an adenovirus expressing TSHR-289 once, and the rate of hyperthyroidism reached 100% at 6 week (17). This model reduced the number of immunizations, thus reducing the modelling time.

Several modifications have been made to improve this modelling method. For example, electroporation was used to enhance the expression of human TSHR *in vivo* and induce hyperthyroidism in mice (24). The deletion of CD4(+) CD25(+) T cells by immune manipulation enhanced disease severity in mice (25). Besides, Kimberly et al. generated mice deficient in STAT6 (26) and STAT4 (27) based on an activator of transcription proteins by gene targeting. The incidence and severity of hyperthyroidism were higher in STAT4-deficient mice than in wild-type and STAT6-deficient mice after immunization with adenovirus containing amino acid residues 1–289 of TSHR (28). In addition, prolonged immunization with repeated injections of adenovirus containing TSHR A subunit appears to induce a more stable Graves phenotype in mice (29).

## Chemically Induced Hyperthyroidism

Non-autoimmune forms of hyperthyroidism are mainly caused by artificial thyroxine supplementation. Exogenous thyroxine supplementation included dissolving levothyroxine in drinking water and intraperitoneal injections (18, 30). Kathrin et al. illustrated that C57BL/6NTac mice receiving intraperitoneal injection of 1 µg/g body weight levothyroxine over 6 weeks exhibited hyperthyroidism (19). Notably, intraperitoneal injections of levothyroxine should not be given more than 48 hours apart. Otherwise, transient hypothyroidism may occur, which may be due to the inhibition of the pituitary-thyroid axis caused by levothyroxine injection in mice (19). Nevertheless, the hyperthyroidism caused by exogenous thyroxine supplementation is temporary, not continuous, and cannot be separated from continuous thyroid hormone supplementation.

## Models of Graves’ Orbitopathy

GO is the most common and severe manifestation of GD and is characterized by orbital inflammation and tissue remodelling (31). Remodelling of the orbital tissue can lead to eye redness, swollen eyes, double vision, and visual impairment (32, 33). One study found that immunizing mice with human TSHR A subunit particles plasmid by close field electroporation resulted in histological signs of orbital lesions, simulating Graves’ ophthalmopathy (34).

Mice immunized with adenovirus carrying the human TSHR A subunit are a very widely used experimental model for GD (35) because of their high incidence and reproducibility. However, whether the method can successfully induce GO is still controversial. Some studies suggest that orbital lesions do not occur after adenovirus TSHR immunization (36). However, the conclusion was based on an analysis of the short-term adenovirus induction protocols. Studies have shown that prolonging the induction time of adenovirus expressing the TSHR (ad-TSHR) A subunit can increase the percentage of successful induction of GO (37). Zhang et al. injected an adenovirus expressing the human TSHR A subunit into the muscle of female BALB/c mice 9 times and successfully induced GO model after long-term ad-TSHR A subunit immunization (37). The frequency of GO in the ad-TSHR A subunit group was 70%, and adipogenesis, lymphocyte infiltration and tissue fibrosis were observed in the long-term animal model (37).

To explore new methods of treating orbital inflammation, new methods of simulating orbital inflammation were attempted. A mouse model of orbital inflammation induced by oxazolone injection outlines some clinical features of thyroid eye disease and other possible features of nonspecific orbital inflammation (38). The model can consistently and repeatedly display clinical, X-ray, and histopathological phenotypes with minimal trauma to ocular or adnexal structures.

Furthermore, Park et al. clarified that when zymosan A was intraperitoneally injected into SKG mice, the eyes of these mice presented with exophthalmos and blepharitis (39). Compared with the control group, orbital adipogenesis and cell infiltration were enhanced, and the concentrations of serum inflammatory factors were increased (39). Furthermore, the browning of orbital adipose tissue may be a potential pathological mechanism accounting for the increase of periorbital adipose tissue. Besides, Park et al. demonstrated that the expression of uncoupling protein 1 increased., which consolidated the browning of adipose tissue, thus leading to increased orbital fat production (39). It was a novel mouse model of the GO-like inflammatory fat phenotype that might be induced by T cell-mediated autoimmune response. This mouse model provides us with the opportunity to investigate the potential molecular mechanisms by which GO enhances adipogenesis and ultimately provides potential therapeutic targets to replace conventional therapy of GO.

## Novel Experimental Therapies of Graves’ Disease in Mouse Model

The initial treatment for GD is usually antithyroid drugs, such as methimazole and propylthiouracil, followed by radioiodine therapy or surgical excision of the thyroid (40). However, these treatments have a relatively high recurrence rate and significant side effects (1). Treating cases of refractory GD and concomitant eye disease/disease is particularly challenging. GO is a major and serious extrathyroidal manifestation of GD. Even mild Graves’ ophthalmopathy can greatly affect the quality of life (31). Severe and active GO is often treated with immunosuppressants, such as glucocorticoids. However, these treatments have many side effects and make patients more susceptible to infection (31). Thus, in recent years, many studies have explored new treatment options for GD and GO to improve patients’ quality of life. In the mouse models of GD, we have identified a number of interventions for the treatment of disease and summarized them in this review (**Table 2**).

**Table 2:** Overview on novel experimental therapies of Graves’ disease and Graves’ orbitopathy.

Therapies	Species	therapeutic effect	Reference
Intraperitoneal injection of diosgenin, 24d	Female BALB/c	GD, relieve goiter formation	(41)
Intravenous Administration of cyclic peptide 836, 6 months	Female BALB/c	GD and GO, improve investigated parameters	(42)
ICAM-1 mAb and siRNA	BALB/c	GD, improved several indexes	(10)
Icariin, intragastrical for 4 weeks	Female BALB/c	GO, inhibit adipogenesis	(43)
Injection hPMSCs into left orbit	Female BALB/c	GO, inhibit adipogenesis	(44)
Fucoxanthin	BALB/c	GO, anti-inflammatory and anti-oxidative	(45)

GD, Graves’ disease; GO, Graves’ orbitopathy; ICAM-1 mAb, monoclonal antibody against intracellular adhesion molecule-1; hPMSCs, human placental mesenchymal stem cells.

Diosgenin (Dio) is a naturally occurring steroid saponin. Studies have shown that Dio can dose-dependently inhibit the proliferation of thyroid cells by suppressing the expression of IGF-1, NF-κB, cyclin D1 and PCNA, thus reducing goiters in GD mouse models (41). Further work leaded to new specific targeted therapies for GD. Studies in the mouse models of GD and GO illustrated that the leucine-rich cyclic peptide 836 simulating the repeat domain of TSHR improved or cured all study parameters after six consecutive months of injections (42). Because the cyclic peptide imitated part of the TSHR, it was presented to the immune system by antigen-presenting cells. Thus, it suppressed the immune response. This might account for the effect of cyclic peptides in the mouse models of GD. In addition, biologically active siRNA targeting TSHR and monoclonal antibodies against intracellular adhesion molecule-1 (ICAM-1 mAb) were proven to significantly improve symptoms and the corresponding hormone and antibody levels in GD mouse models (10). Moreover, ICAM-1 mAb was superior to siRNA targeting TSHR (10). These two therapies have shown potential efficacy as novel therapies for GD and thus might be therapeutic options that could be used in addition to the existing interventions.

As for GO, there have also been many reports on the treatment of GO in recent years. Icariin, a flavonoid separated from plants in the genus *Epimedium*, was found to inhibit the differentiation of preadipocytes *in vitro* and in mouse models of GO. It also possibly regulated the AMPK/mTOR pathway to reduce the expansion of orbital muscle adipocytes and the accumulation of lipid droplets (43). These results revealed the possible protective mechanism of icariin and suggested that icariin may be a novel therapeutic candidate for the prevention and treatment of GO. Furthermore, researchers found that the injection of human placental mesenchymal stem cells (hPMSCs) into the orbit of mice model of GO could suppress lipid accumulation and the corresponding activators, thereby inhibiting orbital fibrosis and adipogenesis (44). In addition, hPMSCs can improve orbital adipogenesis in the model (44). The study demonstrated that hPMSCs might restore pathogenic activation of orbital fibroblasts in experimental models of GO and confirmed the feasibility of hPMSCs as a novel treatment for GO patients. Additionally, it has been reported that fucoxanthin, a carotenoid, can inhibit 1L-17 mRNA expression to produce anti-inflammatory and antioxidant effects in GO mouse model (45). However, the therapeutic effects of fucoxanthin remain to be further studied.

## Antigen-Specific Therapy to Induce Tolerance

In recent years, studies have utilized mice immunized with ad-TSHR to study antigen-induced tolerance against GD. Misharin et al. suggested that a single injection of the glycosylated TSHR A subunit prior to TSHR immunization protected mice from GD. The effect was antigen-specific and lasted for a short period of time (8). Similarly, Wu et al. expounded in their study that neonatal mice pre-treated with adenovirus expressing the TSHR A subunit at high doses could developed significant resistance to GD (46). However, although the TSHR A subunit protein is an underlying immunomodulator, it cannot be applied to reverse hyperthyroidism in mice. Additionally, it is unlikely to be utilized to prevent further progression in patients that have already been diagnosed with GD. Based on these studies, the immune tolerance model provides hope for the prevention of GD. In susceptible populations, it is expected that the TSHR A subunit protein vaccine may induce resistance to GD, and these populations could develop long-term protection against the progression of GD.

## Conclusions

In conclusion, a long-term disease model of GD that stably presents with the characteristics of the human disease has been established. However, all animal models, especially those experimentally induced, have limitations. Studying the same disease in multiple models can provide greater insights than studies in a single model.

Current treatments for autoimmune diseases include inducing immune tolerance through TSHR-specific antigens and several experimental therapies. This topic requires further research but holds great promise as a possible alternative to traditional treatments.

## Author Contributions

All listed authors have made substantial, intellectual and direct contributions to the work and approved its publication.

## Funding

The study was supported by the Shanghai Natural Science Foundation, under Grant number 21ZR1449600.

## Conflict of Interest

The authors declare that the research was conducted in the absence of any commercial or financial relationships that could be construed as a potential conflict of interest.

## Publisher’s Note

All claims expressed in this article are solely those of the authors and do not necessarily represent those of their affiliated organizations, or those of the publisher, the editors and the reviewers. Any product that may be evaluated in this article, or claim that may be made by its manufacturer, is not guaranteed or endorsed by the publisher.
